# The relevance of acute kidney injury in dehydrated children

**DOI:** 10.3389/fped.2025.1698954

**Published:** 2025-12-09

**Authors:** Dovilė Ruzgienė, Ernestas Viršilas, Augustina Jankauskienė

**Affiliations:** Clinic of Children’s Diseases, Institute of Clinical Medicine, Faculty of Medicine, Vilnius University, Vilnius, Lithuania

**Keywords:** dehydration, volume depletion, acute kidney injury, children, outcomes

## Abstract

**Introduction:**

Dehydration is the leading cause of acute kidney injury in children, yet its true burden and clinical relevance in the general pediatric population remain poorly characterized. The majority of existing research on pediatric acute kidney injury primarily focuses on cases occurring after cardiac surgery or in critically ill children, leaving dehydration-associated AKI understudied.

**Methods:**

A PRISMA-guided systematic search of PubMed and EBSCO databases was conducted to identify studies published from 2010 to 2025 investigating pediatric acute kidney injury due to dehydration. The risk of bias was assessed using the Newcastle-Ottawa Scale for observational studies and the modified Cochrane Collaboration Risk of Bias 2.0 tool for randomized controlled trials. Outcomes examined included incidence, severity, short-term and long-term effects of acute kidney injury, and risk factors.

**Results:**

Out of 2,251 records, 31 studies met the inclusion criteria. Most were cohort or case-control studies of good quality, while one randomized controlled trial was identified as having a low risk of bias. The incidence of acute kidney injury varied widely, with higher rates observed among younger children. Hypovolemia, sepsis and shock were key risk factors for the development of acute kidney injury. Stage I acute kidney injury was the most commonly reported stage. Short-term outcomes included kidney recovery, mortality, and hospitalization metrics; long-term data were scarce, primarily focusing on kidney function preservation.

**Conclusions:**

Prerenal acute kidney injury dominates in high-risk pediatric settings. Most pediatric acute kidney injury cases appear to be unifactorial in origin. Further long-term follow-up studies are necessary to better understand the enduring effects of acute kidney injury in children and its potential progression to chronic kidney disease.

## 1 Introduction

Acute kidney injury (AKI) in the pediatric population is primarily attributable to kidney hypoperfusion, often resulting from dehydration or other volume-depleting conditions (1). Dehydration is a prevalent etiological factor in pediatric AKI, with acute gastroenteritis representing the primary underlying cause. The pathophysiological mechanism involves kidney hypoperfusion, which accounts for approximately 12% of AKI cases within the pediatric population (2). AKI resulting from this pathophysiological mechanism is referred to as prerenal AKI. Alternatively, a more recent term, decreased renal perfusion, may be used synonymously. Current research predominantly concentrates on AKI occurrences following cardiac surgery and among critically ill children admitted to PICUs (3–6). However, investigations specifically addressing pediatric AKI precipitated by dehydration remain limited.

Children who experience AKI are at increased risk for extended hospital stays, progression to chronic kidney disease (CKD), and the development of arterial hypertension (AH) (7–10). CKD exerts profound effects on pediatric physical and psychological health, impairs growth and neurocognitive development, diminishes quality of life, and elevates the risk of early cardiovascular morbidity and mortality (11–13). Therefore, early identification of AKI and its associated risk factors is vital for improving clinical outcomes in this vulnerable population. Enhanced awareness and timely intervention may mitigate short-term and long-term complications and promote better prognostic trajectories.

The aim of this study is to systematically review the existing literature concerning dehydration and its association with AKI in pediatric population. The review focuses on examining key characteristics, including the incidence of AKI, identified risk factors, and both short-term and long-term clinical outcomes related to pediatric AKI.

## 2 Materials and methods

### 2.1 Study design

This systematic review was conducted strictly according to PRISMA-2020 (Preferred Reporting Items for Systematic Reviews and Meta-Analyses) guidelines and was prospectively registered in PROSPERO (International Prospective Register of Systematic Reviews; registration number of CRD420251003377).

### 2.2 Search strategy

The relevant studies were systematically searched in the PubMed and EBSCO databases by two independent reviewers (D.R. and E.V.). The search covered all the literature that was published from 2010 to April 2025, with no language or regional restrictions. The search strategy used in PubMed was as follows: ((children OR pediatric OR teenager) AND (risk factors) AND (dehydration OR volume depletion OR hypovolemia) AND (causes) AND (acute kidney injury OR acute renal failure OR acute renal injury OR AKI OR acute kidney insufficiency)). A double search strategy was implied using MESH terminology and without (manual screening). However, in the manual screening process, a reviewer systematically screened all 2,251 records and excluded articles that did not meet the inclusion criteria. The filters of “Clinical Trial”, “Comparative Study”, “Multicenter Study”, “Observational Study”, “Humans”, “Child: birth-18 years”, “exclude preprints”, “year 2010–2025” were applied. The search conducted in the EBSCO database utilized the same keywords as those used in PubMed. Due to differences in the available filtering options across databases, the following filters were applied in EBSCO: “01/01/2010–04/30/2025”, “Database”, “Academic journal”, “Country Report”, “Educational Report”, “Report”, “Article”, “Journal article”, “Research”, “Full text”, “References Available”, “Peer Reviewed”, “SmartText Searching”, “Apply equivalent subjects”. The search yielded 254 articles, all of which were manually screened based on the predefined inclusion criteria. While both approaches yielded similar results, manual search had 1 additional publication (14), which was missed while using MeSH terminology. For brevity and precision, only the MeSH representation of the search strategy is depicted.

### 2.3 Study selection

Duplicate articles were removed from the dataset. Literature reviews and clinical cases were excluded at the initial screening stage. Titles and abstracts were screened to identify initial studies that complied with the predefined inclusion criteria:

- Children from 1 month to 17 years;
- AKI (as diagnosed using any recognized diagnostic criteria) and dehydration/hypovolemia.

The exclusion criteria involved the following:

- Infants (less than 1 month of age);
- Adults (over 17 years of age);
- Children having AKI of renal and post-renal etiology;
- Children with CKD.

Studies including individuals outside the specified age range (<1 month or >17 years) were retained if they contained relevant data for the population of interest. Subsequently, the full texts of all potentially eligible studies were retrieved, downloaded, and reviewed to confirm their eligibility according to the same criteria. The selected studies were imported into Zotero for organization and reference management. Two reviewers (D.R. and E.V.) independently conducted the literature screening described above. Any discrepancies in study selection were resolved through discussion until a consensus was reached. A third reviewer (A.J.) arbitrated disagreements when necessary.

### 2.4 Data extraction

Data extraction from each eligible study was performed by two reviewers independently. The main outcomes analyzed were as follows:

- The incidence of AKI in dehydrated children;
- The severity of AKI in dehydrated children;
- The short-term and long-term effects of AKI in dehydrated children.
- Additional analyzed outcomes were:
- Risk factors associated with AKI incidence;
- Risk factors associated with AKI severity.

Some differences in the collected information —references (authors, year of publication, country), study design, sample size, analyzed outcomes, and results —were observed at the end of data extraction. The missing or unclear data were not included in the analysis.

### 2.5 Quality assessment

The assessment of study quality was performed by two independent reviewers (D.R. and E.V.) using the modified Cochrane Risk of Bias 2.0 (RoB 2) tool (version 2, Cochrane Collaboration, London, UK) for randomized controlled trials, and the Newcastle-Ottawa Scale (NOS) for non-randomized studies, including case-control and cohort studies. According to the RoB 2 criteria, the risk of bias across all domains—the randomization process, deviations from the intended interventions, missing outcome data, measurement of the outcome, and selection of the reported result—was categorized as low, some concerns, or high risk of bias. The NOS evaluates studies based on three key domains: patient selection, comparability of groups, and outcome assessment for cohort studies; and patient selection, comparability, and exposure assessment for case-control studies. These domains determined the overall risk of bias in non-randomized studies, and study quality was rated as good, fair, or poor. Discrepancies between the two reviewers (D.R. and E.V.) were resolved through discussion, with a third reviewer (A.J.) involved as needed. Heterogeneity among studies was assessed, where applicable, using Cochran's *Q* test and quantified with the I² statistic, a random-effects model (Restricted Maximum Likelihood estimator) was applied, given the expected methodological and clinical diversity between studies.

## 3 Results

### 3.1 Study selection

**Figure 1:**
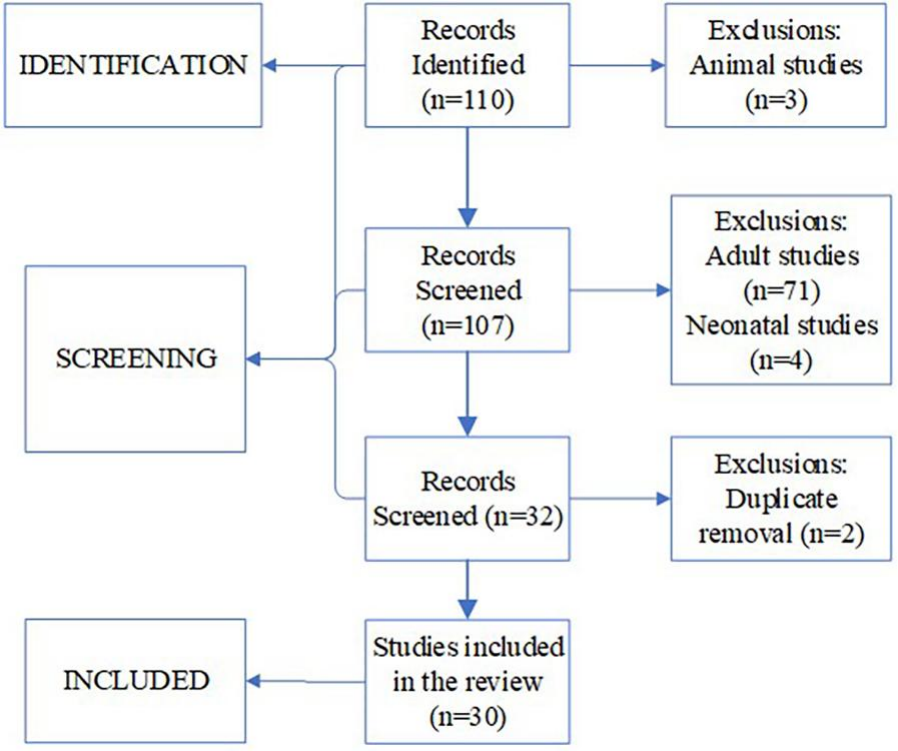
Search strategy flowchart.

The initial MeSH search yielded 110 results. Studies older than 2010, adult studies as well as animal studies were rejected. After removing duplicate entries, 30 publications were selected for the final list (Figure 1). The manually found publication was added to the final list, bringing the total to 31 publications.

### 3.2 Study characteristics

The majority of the included publications were observational studies, several case-control studies with only one study identified as randomized controlled trial (RCT). Most of the publications had a specific demographics of participants, such as children with diabetic ketoacidosis (DKA) (14, 15), dehydration (16–20), acute gastroenteritis (21), while others investigated risk factors, etiology, incidence and the overall outcomes of AKI. Table 1 summarizes the study characteristics and patient demographics reported in each publication. Significant heterogeneity was observed across the included studies (*I*² = 67%, *Q* = 49.32, *p* < 0.001), indicating substantial variability.

**Table 1:** Study characteristics and patient demographics.

Study	Year	Country	Study type	Children demographic	Study aims	AKI definition	Incidence (if applicable)	Total Enrolled/AKI patients	Outcomes analyzed	Key findings
Parikh et al. (27)	2021	India	Observational cohort	1 month—12 years	Incidence, severity and risk factors of AKI among PICU patients.	PRIFLE max	73.2%	*n* = 220/161	Kidney replacement therapy (KRT), Mortality, Length of hospital and PICU stay.	Very high incidence with several risk factors were identified. Main risk factors for AKI: infancy, hypovolemia, shock, sepsis.
Xin Xu et al. (37)	2018	China	Observational cohort	0–18 years	Estimate the incidence of pediatric AKI and its' risk factors.	KDIGO	20.0%	*n* = 1,01,836/19,908	Mortality, Length of hospital stay, Daily cost of hospitalization, Kidney function recovery.	Cardiac surgery or congenital heart disease was the biggest risk factor for AKI development.
Marzuillo et al. (22)	2021	Italy	Observational cohort	Unspecified	To assess the prevalence and risk factors of AKI in children hospitalized with acute gastroenteritis.	KDIGO without urine output	-	*n* = 114/28	KRT, Length of hospital stay.	Quarter of acute gastroenteritis hospitalizations involve AKI, with longer stays linked to greater severity.
Anigilaje et al. (28)	2019	Nigeria	Observational cohort	1 month—15 years	Reporting etiology, clinical profile and short-term outcomes of AKI amongst tertiary center patients in Nigeria.	pRIFLE	-	*n* = 1,634/43	KRT, Mortality, Long-term kidney function.	The most common etiologies of AKI were sepsis, acute glomerulonephritis (GN), diarrheal dehydration and haemolytic uremic syndrome (HUS). Male gender and pulmonary edema significantly increase mortality.
Chisavu et al. (38)	2023	Romania	Observational cohort	0–18 years	Incidence of AKI and awareness of AKI amongst physicians.	KDIGO	1.65%	*n* = 1,28,036/2,194	Mortality, Length of hospital stay.	High incidence rate of AKI. The most frequent was pre-renal AKI. AKI awareness was low amongst physicians in Romania.
De Zan et al. (26)	2020	Italy	Observational cohort	0–20 years	Incidence, risk factors and clinical outcome of AKI among PICU patients.	KDIGO	27% (among PICU patients)	*n* = 811/222	KRT, Mortality, Length of PICU stay, Kidney function recovery.	Incidence and mortality is high among AKI PICU patients. Risk factors are mainly associated with illness severity.
Sun et al. (25)	2024	China	Cross sectional survey	0–14 years	Incidence, misdiagnosis rate, main causes, medical costs of AKI.	Custom made, similar to KDIGO	4.34%	*n* = 29,639/1,286	Mortality, Length of hospital stay, Daily cost of hospitalization, Kidney function recovery.	AKI is not uncommon in hospitalized children and frequently underrecognized. AKI increases hospitalization costs and risk of mortality.
Huang et al. (88)	2020	Taiwan	Observational cohort	0–18 years patients with DKA	To determine the incidence, prevalence, severity, and clinical correlates AKI in DKA. Evaluate association between AKI severity and recovery time from metabolic acidosis.	KDIGO	-	*n* = 223/170	-	AKI occurred in 56.5% of DKA episodes (18% severe) and was strongly associated with dehydration indicators More severe AKI may lead to increased recovery time. Most children (79.9%) recovered from acidosis within 24 h, regardless of AKI severity.
Van Driest et al. (14)	2020	USA	RCT	1 month—21 years	To assess whether AKI risk alerts increase AKI screening.	KDIGO	-	*n* = 12,731/1,084	Mortality, Length of hospital stay.	The implementation of AKI alerts did not lead to improved diagnostic yield, as existing diagnostic tools were already effective.
Shahrin et al. (50)	2020	Bangladesh	Observational cohort	0–12 months	Describe the clinical and laboratory characteristics and associated features AKI in infants with diarrhea.	Custom made, Creatinine and Urine output based	-	*n* = 731/146	-	Several predicting factors for AKI among infants with diarrhea. Dehydration, sepsis and hypernatremia were found to be associated with AKI among infants with diarrhea.
Tanwar et al. (17)	2024	India	Observational cohort	1–2 months of age	Evaluate the clinical profile and outcome in the pediatric emergency department with hypernatremic dehydration.	KDIGO	-	*n* = 55/48	KRT, Mortality.	Acute kidney injury stage 3, shock, and need for ventilation are associated with poor outcome in infants with hypernatremic dehydration.
Duzova et al. (2)	2010	Turkey	Observational cohort	1 month—18 years	Characterize etiology and clinical features of AKI and identify associated prognostic factors.	pRIFLE	-	*n* = 472/472	KRT, Mortality.	Evaluated risk factors for AKI mortality in Turkey population. Major risk factors for AKI were mechanical ventilation, congenital heart disease, hypervolemia and metabolic acidosis in newborns; mechanical ventilation, hypoxia and intrinsic AKI in children >1 month.
Çelik et al. (89)	2013	Unspecified	Case control study	Unspecified	Evaluate plasma and urine Neutrophil Gelatinase-Associated Lipocalin (NGAL) levels as early biomarkers of prerenal AKI mildly to moderately dehydrated children.	Unspecified	-	*n* = 30/(unspecified)	-	Mildly or moderately dehydrated children have higher plasma and urine NGAL concentrations in early AKI stages compared to creatinine levels.
Louzada et al. (29)	2021	Unspecified	Observational cohort	0–17 years	Evaluate prevalence, risk factors and outcome of AKI among PICU population in a tertiary care university hospital.	KDIGO or pRIFLE or AKIN or Acute kidney injury work group	-	*n* = 1,131/143	KRT, Mortality, Length of hospital and PICU stay, Long-term kidney function.	Estimated AKI prevalence was 12.6%—12.9% depending on the diagnostic criteria used. Sepsis, need for ventilation and use of vasoactive medications increased risk for AKI.
Marzuillo et al. (20)	2022	Unspecified	Case control study	Unspecified	Evaluation of heart rate variability as a predictor of acute kidney injury and dehydration in acute setting.	KDIGO	-	*n* = 185/81	-	Heart rate variability can be a predictor of AKI.
Marzuillo et al. (19)	2024	Italy	Observational cohort	0–18 years	Validation of heart rate variability as a predictor of acute kidney injury and dehydration in pediatric emergency department.	Custom serum creatinine/basal creatinine (HC/BC) ratio	-	*n* = 256/50	-	Heart rate variability cutoffs could be used as predictor for AKI. However, it remains nonspecific finding.
Balestracci et al. (21)	2015	Argentina	Case-control study	1 month—18 years	Estimate the prevalence of ibuprofen-associated AKI, assess its role as a risk factor, and evaluate the impact of dehydration severity on its development.	pRIFLE	-	*n* = 105/46	KRT, Kidney function recovery.	Ibuprofen exposure found to be considerable risk factor for AKI in dehydrated children.
Tas et al. (15)	2024	Unspecified	Observational cohort	1–18 years patients with DKA	Determine hyperchloremia association with an increased risk of AKI in patients with diabetic ketoacidosis.	pRIFLE	-	*n* = 113/22	KRT, Length of hospital stay.	Hyperchloremia was not associated with AKI; AKI development was linked to the volume of iatrogenically administered fluids, irrespective of their type.
Shireen et al. (18)	2021	Bangladesh	Observational cohort	1 month—6,5 years with AKI	Assess the safety of varying amounts of dextrose in normal saline with various dilutions of 3% NaCl and intermittent peritoneal dialysis in children with severe hypernatremic dehydration and AKI.	pRIFLE	-	*n* = 45/45	Mortality.	Management of severe hypernatremic dehydration with AKI by intermittent peritoneal dialysis (PD) and with various dilutions of intravenous (IV) fluid containing sodium concentration around 10 mEql/L lower than patient's serum is safe.
Feehally (30)	2016	International	Cross-sectional study	Children and adults	Provide information about world-wide epidemiology and causes of AKI.	KDIGO	-	*n* = 354/354 (children)	KRT, Mortality.	Hypotension and dehydration were the most common causes of AKI. Mortality was higher in low and lower middle-income countries.
Ahmed et al. (16)	2022	Egypt	Cross-sectional study	1–13 years pediatric patients with DKA	Evaluation of the association between hyperchloremia and AKI in pediatric DKA patients.	pRIFLE	-	*n* = 70/20	Albuminuria in a long-term.	Microalbuminuria and hyperchloremia have stat. significant associations with AKI.
Paudel et al. (86)	2021	Nepal	Observational cohort	2 months—14 years	Evaluation of the incidence, etiology and short-term outcome of AKI in a tertiary centre.	pRIFLE	5.9% (18.23% among PICU patients)	*n* = 942/56	KRT, Mortality, Length of hospital stay, Kidney function recovery.	The incidence of AKI was high in pediatric patients; AKI increased the length of hospital stay and mortality.
Ashish et al. (31)	2023	India	Observational cohort	2 months—12 years	Estimation of the frequency, etiology, outcome and risk factors for mortality in community-acquired AKI	KDIGO	-	*n* = 2,780/215	KRT, Mortality, Length of hospital stay, Kidney function recovery, Long-term kidney function.	The majority of community-acquired AKI resulted from diarrheal diseases and sepsis. Long-term follow-up is required because of the high risk of progression to CKD.
Soltysiak et al. (41)	2023	Poland	Case-control study	0–18 years	Determine AKI proportion in pediatric DKA patients	KDIGO	-	*n* = 198/27	Long-term kidney function	14% of DKA patients developed AKI. AKI and repeated DKA potentialy hastens diabetic kidney disease
Batte et al. (39)	2022	Uganda	Observational cohort	2–18 years	Determine AKI incidence in pediatric sickle cell anemia (SCA) patients	KDIGO	-	*n* = 185/90	Mortality.	47% of SCA patients developed AKI, with the majority of episodes consisting of Stage 1. Hepatomegaly, hypovolemia, and infection were identified as independent risk factors.
Halle et al. (32)	2017	Cameroon	Observational cohort	0–17 years	To report the epidemiological profile and outcome of kidney failure among hospitalized children in the main tertiary referral hospital.	pRIFLE	-	*n* = 103/87	KRT, Kidney function recovery, mortality.	The most frequent reasons for AKI were acute tubular necrosis and pre-renal AKI. Majority of patients had most severe AKI stage (F). AKI determined a high mortality in patients due to limited access to specialized paediatric nephrology services in the country.
Chisavu et al. (33)	2023	Romania	Observational cohort	0–18 years	To determine the relationship between AKI duration and severity and progression to CKD	KDIGO	1.64%	*n* = 2,765/2,346	KRT, Mortality, Kidney function recovery, Long-term kidney function.	Prerenal AKI was the most frequent. The continuum of AKI resulted in an increased new-onset CKD. CKD progression occured in 4% of patients with a 5.3-fold increase in acute kidney disease patients.
Rustagi et al. (34)	2016	India	Case-control study	2 months—18 years	To predict AKI and its outcome in critically ill children, and to evaluate the associated risk factors.	RIFLE	14% (among PICU patients)	*n* = 380/53	KRT, Mortality, Length of hospital stay.	Patients with AKI had 4.5-fold higher mortality than those without. The most common diagnoses underlying AKI were acute lower respiratory tract infection, CNS illness, and severe dehydration. The RIFLE criterion may help early identify patients at risk for AKI. Age <5 years, shock, infection, multiorgan dysfunction, PRISM score of >10 and hypoalbuminemia were identified as independent risk factors.
Nawaz et al. (35)	2018	India	Case-control study	3 months—12 years	To determine the incidence and clinical profile of AKI in hospitalized children using AKIN criteria.	AKIN	25.2%	*n* = 600/151	KRT, Mortality, Length of hospital stay, Long-term kidney function.	Patients with AKI had longer hospital stays and a higher mortality rate. Stage 1 AKI was the most prominent stage in the study. Nephrotoxic drugs, hypovolemia, sepsis, acidosis and mechanical ventilation were independent predictors for AKI.
Tresa et al. (36)	2017	Pakistan	Observational cohort	1 month—15 years	To determine the etiology, clinical profile, and short-term outcome of pediatric AKI at tertiary care hospital in Pakistan	pRIFLE	-	*n* = 116/116	KRT, Mortality, Kidney function recovery.	Most AKI cases were attributed to intrinsic renal causes, whereas prerenal etiology accounted for the smallest proportion (9.5%). The majority (76.7%) AKI were of Failure stage. Most of the patients recovered (58.6%), but severe cases frequently progressed to CKD/ESRD.

### 3.3 Quality assessment

**Figure 2:**
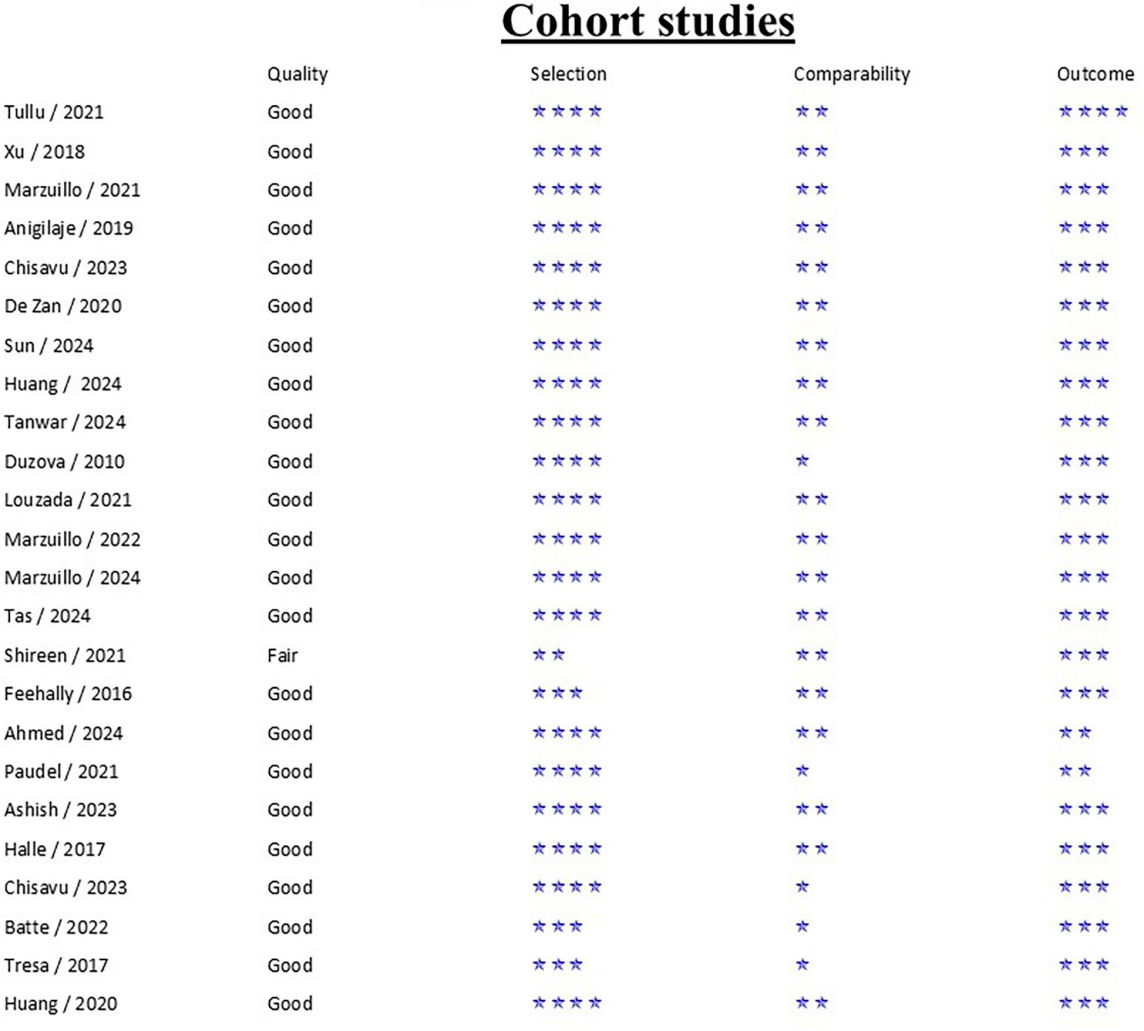
Risk of bias assessment using NOS—cohort studies (2, 14–19, 21–29, 32, 33).

**Figure 3:**
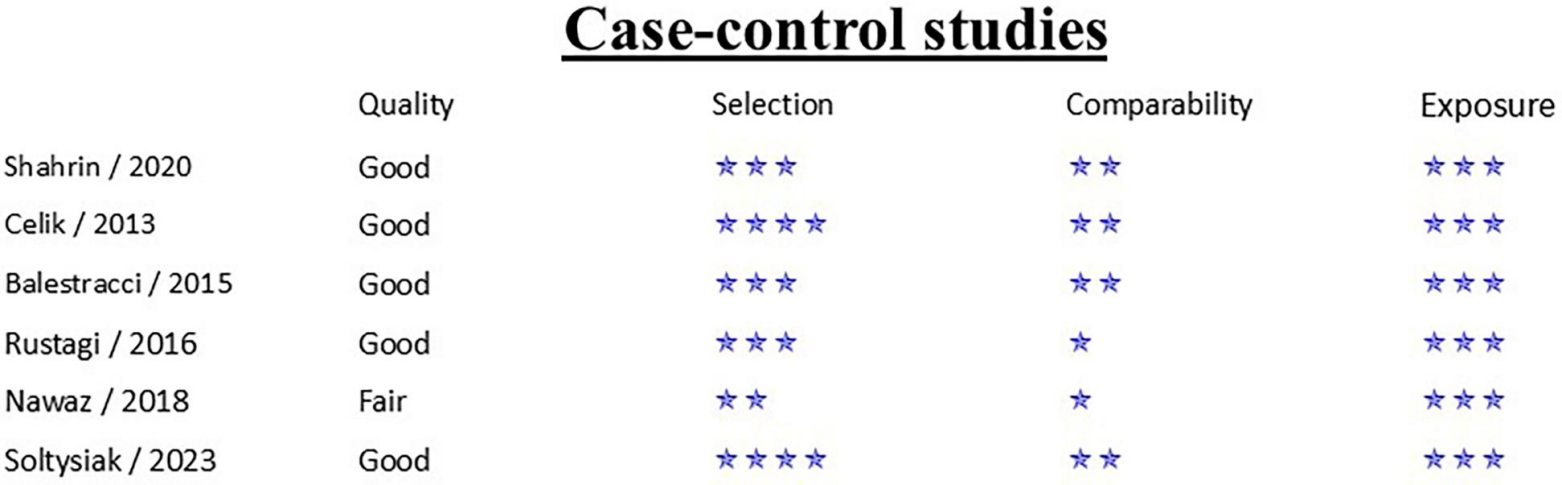
Risk of bias assessment using NOS—case-control studies (20, 30, 31).

**Figure 4:**
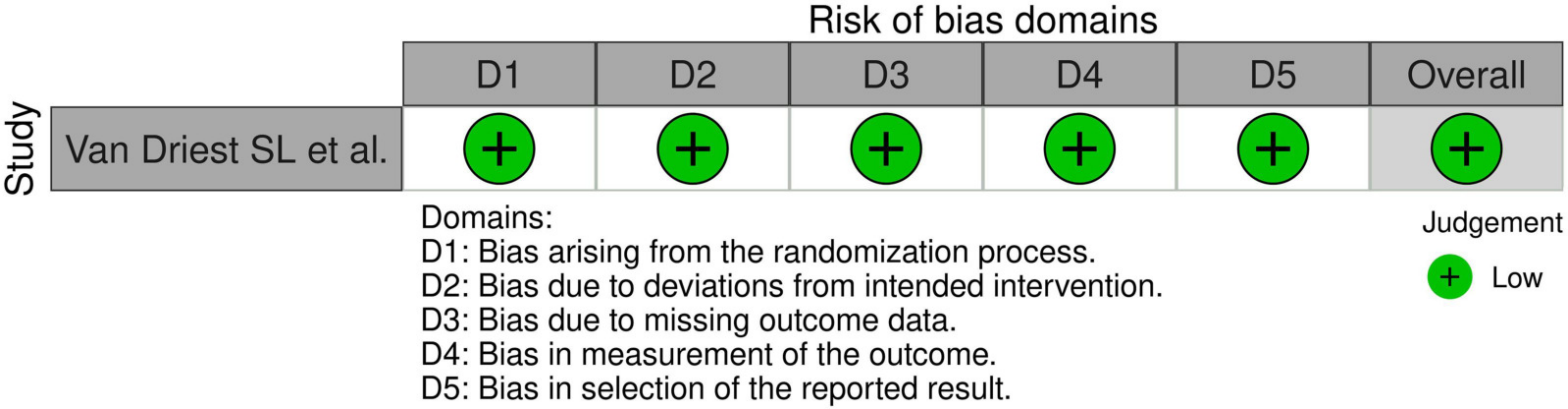
Risk of bias assessment using the modified RoB 2.0 tool (29).

Between observational and case control studies, NOS scale median was 8 with an interquartile range (IQR) of 1, with the lowest score of six. RCT was evaluated as a low risk of bias according to the ROB2 criteria. Detailed results regarding the risk of bias in the included studies are presented in Figures 2–4.

### 3.4 AKI incidence, severity, outcomes and risk factors of dehydrated children

As expected, the reported incidence of AKI in the literature varied widely, ranging from as low as 1.65% to as high as 73.2%, smaller children and infants had higher rates of incidence than older children (23, 24). One third of the studies assessed and reported the severity of AKI, the majority of them focused on the detection of AKI as a sequela of prerenal etiology. In contrast, a minority targeted the AKI event itself and its staging. Several studies assessed the most prominent risk factors associated with the prerenal cause of AKI. Few of the studies (2) focused on risk factors for mortality, rather than for AKI *per se*. The risk factors for AKI that were analyzed in the studies are presented in Table 2. AKI severity was reported in several studies, most of which employed a three-stage classification system to describe the extent of kidney injury (15, 26, 27, 29). As expected, stage 1 was the most prevalent, ranging from 39.0–70.8%, with stage 2 and 3 being less common, ranging from 24.0–33.1% and 5.8%–37%, respectively. The distribution of AKI staging appeared to be closely related to study sample size — larger studies tended to report a higher proportion of early-stage AKI, whereas smaller studies showed more heterogeneous staging patterns. The main analyzed short-term outcomes were kidney replacement therapy (KRT) (2, 16, 17, 21, 22, 24, 26, 27, 29, 32, 33), mortality (2, 16, 17, 22–27, 29, 32, 33), the length of stay in hospital (14, 16, 21–23, 25–27, 29, 32), and PICUs (20, 22, 26, 32), daily cost of hospitalization (23, 27), and kidney function recovery (21, 25, 26, 30, 32, 33, 36, 40). Long-term outcomes focused on kidney function preservation, including assessments of glomerular filtration rate (GFR) and albuminuria (16, 28, 29, 32, 33, 35, 41). The precise description of the short- and long-term outcomes of AKI analyzed in each study is shown in Table 1.

**Table 2:** The risk factors for AKI that were analyzed in the studies.

Category	Risk factor	The number of studies identifying the factor
Disease-related risk factors		
	Diabetes/Diabetic ketoacidosis	2
	Infection/Sepsis	5
	Shock	5
	Diarrhea/dehydration/hypovolemia	7
	Urinary tract obstruction	1
	Intestinal obstruction	1
	Glomerulonephritis	1
	Hypoxic ischemic encephalopathy	1
	Hemolytic uremic syndrome	1
	Multi-organ dysfunction	1
	PRISM score >10	1
Laboratory-related risk factors		
	Low serum bicarbonate level	1
	Hypoalbuminemia	1
	Thrombocytopenia	1
	Acidosis	3
	Hyperchloremia	1
Patient-related risk factors		
	Mechanical ventilation	3
	Nephrotoxic drugs	1
	Age	2

## 4 Discussion

### 4.1 Incidence

As is often the case in pediatric research, the lack of standardized definitions of AKI contributes to wide variability in reported incidence rates. In our analysis, this variability was further compounded by differences in age-based inclusion criteria across studies, which ranged from as young as 0 or 1 month to upper age limits of 12 (22), 14 (27), 18 (23, 25), and even 20 (26) years. Although KDIGO guidelines (34) are considered more sensitive to AKI detection, in our review the highest incidence was detected using pRIFLE criteria (35). Only one of the studies (32) which reached final analysis incorporated AKIN framework (36) for AKI identification. It is widely recognized that studies with lower age cutoffs for inclusion tend to report higher AKI incidence rates. The findings of Parikh et al. exemplify this trend. (22), which reported a remarkably high incidence exceeding 70%. This study was conducted in the PICU setting, where AKI tends to be more prevalent, particularly among patients with more critical conditions or illness severity. Furthermore, while different definitions are known to produce varying degrees of diagnostic sensitivity (37), the authors do not believe it was the cause in this particular review, but rather, variance in age inclusion, country, and setting (PICU, Cardio surgery/congenital heart disease, or general wards) likely accounts for the varying differences detected. Heterogeneity among studies was substantial (*I*² = 67%). This was expected by the authors, given the wide range of AKI definitions, clinical settings, as well as countries in which incidence was assessed.

### 4.2 Risk factors

Most of the general risk factors are already widely available and known (38), even before the dawn of term Acute Kidney Injury, which later became standard and still is today. Some of the studies analyzed or included less commonly assessed factors such as hyperchloremia, microalbuminuria, and heart rate variability (14, 15, 18, 19, 21). Sepsis and shock were prominent risk factors of AKI (22–24), with hazard ratio of 1.69 and 2.81, respectively. These results are consistent with observed in adult counterparts (39), except for hypotension/shock, which doesn't seem to be as detrimental in adults as in pediatric patients (39, 40). Conversely, in adult population, hypertension is recognized as an independent risk factor for both the occurrence and severity of AKI. In contrast, pediatric patients who develop AKI rarely have pre-existing conditions associated with elevated blood pressure. Opposite to the children, for adults it is not uncommon to have hypertension and hyperchloremia concurrently, with animal studies suggesting that both factors synergistic interaction may result in greater harm compared to isolated presence (44). The role of isolated hyperchloremia remains debated—whether it constitutes an independent risk factor for AKI or is a mere reflection of an underlying kidney dysfunction. Two studies examined this question, each arriving at a different conclusion (14, 15).

Hypovolemia was the most commonly identified independent risk factor for prerenal AKI found in 7 studies (2, 17, 27, 28, 35, 39, 50). This finding is not unexpected, as intravascular hypovolemia directly impacts kidney perfusion. Prolonged hypoperfusion leads to acute tubular necrosis, often a principal contributor to prerenal AKI (51). Dehydration, arising from gastroenteritis or associated with elevated serum chloride levels, was frequently cited as a major contributing factor of either the risk for AKI or the direct cause of it (14–16, 21–24). This is not contrary to adult literature, where dehydration (also, commonly, as a result of gastroenteritis) negatively impacts kidney function, especially in older individuals (41–43). Furthermore, a growing body of evidence suggests that chronic low degree repetitive dehydration might lead to CKD (44–46). It appears, the problem is the degree of exposure—if large and prolonged, it results in AKI, while more minor and repetitive dehydration/rehydration cycles hasten CKD. In literature, a large multicenter RCT recently reported that administering normal saline vs. less chloride enriched alternatives (e.g., PlasmaLyte) resulted in higher AKI incidence (47). Several other smaller studies also reached similar implications (48, 49). Nonetheless, the results were met with scrutiny, with experts questioning the strength of the association and its clinical implications (50). In adults as well, the causal relationship between hyperchloremia and AKI remains unresolved and still lacks definitive evidence—mirroring the “chicken or the egg” dilemma (51–56, 65, 66).

Many other conditions closely intertwine with intravascular hypovolemia, severe infection, sepsis (with or without shock), as well as acidosis. Moreover, due to compensatory sodium and chloride retention, hyperchloremia is often encountered as a side effect, especially when administered fluids containing supraphysiological chloride amounts (as in normal saline) which is sometimes mentioned as a risk factor *per se* (63, 67). In patients with severe infection, particularly sepsis, who are already in a hypovolemic state, endothelial dysfunction resulting from microcirculatory disturbance and the systemic inflammatory response further contribute to kidney damage (68, 69). There are some emerging insights and ongoing clinical trials for treatment/management for sepsis induced AKI aimed at mitigating inflammation-mediated damage, however, the current mainstay of management remains appropriate antimicrobial therapy, adequate fluid resuscitation, inotropic support and timely initiation of KRT when indicated (70, 71). Acidosis appeared frequently in our review as a possible contributor to AKI, yet its causal role remains poorly defined. By contrast, its involvement in CKD progression is much better characterized and (72, 73) the underlying pathophysiological mechanism is quite prominent (74). Beyond being a consequence of reduced kidney function, acidosis also acts as a destructive agent in CKD, most likely through the metabolic derangements it triggers.

In our review, mechanical ventilation was also identified as a risk factor for AKI. Historically, the need for ventilatory support was considered merely a reflection of illness severity rather than a direct contributor to AKI. Traditionally, it was (and sometimes still is) employed to reduce the patient's metabolic demand and/or support recovery during critical illness. However, in recent years, especially during COVID19 pandemic, mechanical ventilation became notorious for ventilator induced lung injury (VILI), and accumulating data suggest that its adverse effects extend beyond the lungs, contributing to what is increasingly recognized as ventilator-induced kidney injury (VIKI) (75–77). Proficiency with ventilation is required to selectively adjust settings that usually need to be tailored to specific patient needs, in order not to reinforce the main Lung-Kidney crosstalk damaging components: hypoxia/hypercapnia, cardiac output and venous return. All three of the aforementioned can be significantly affected by ventilation settings (75).

DKA is another risk factor met in our analysis. However, it remains unclear whether hyperglycemia resulting from insulin deficiency is an independent contributing factor, as findings across studies vary. Nonetheless, DKA state commonly causes dehydration/hypovolemia, acidosis and hyperchloremia, two of which were proven to be independent risk factors met frequently in our analysis. Glucosuria is also sometimes encountered with interstitial glomerulonephritis (78, 79). DKA appears to create and promote the intertwining of independent risk factors that collectively contribute to kidney injury, although the damage is not a direct consequence of DKA itself. Additionally, the degree of damage it causes also appears to be associated with DKA length, with prolonged and more dehydrated individuals seem to be especially prone to AKI (57–59, 80, 81).

### 4.3 Outcomes

The incidence of KRT utilization exhibited considerable variability, with rates spanning from 0% in two studies (16, 21), to as high as 67.4% (24). This substantial range is likely attributable to the differences in sample sizes among the studies.

Several studies have investigated the risk factors associated with mortality resulting from AKI. In multivariate analyses conducted by Anigilaje et al., male gender and the presence of pulmonary edema were identified as significant predictors of mortality (24). Parikh et al. further elucidated that young age (*P* = 0.006), hypovolemia (*P* = 0.002), exposure to nephrotoxic drugs (*P* = 0.005), and thrombocytopenia (*P* = 0.001) were significant determinants of increased mortality in pediatric patients with AKI (22). Nawaz et al. identified acidosis, mechanical ventilation, shock and sepsis as independent risk factors for mortality in AKI (35). In the adult population, the primary and most significant predictor of mortality among AKI patients appears to be a higher AKI stage (60, 61). An investigation of the association between treatment modalities and mortality rates indicated that patients undergoing dialysis exhibited a statistically significant increase in mortality (24, 32, 33). This elevated mortality risk may be due to the severity of underlying kidney injury, compounded by the inherent risks associated with KRT. Mortality among AKI patients varies widely, from 5.3% (2) to a 79-fold increase compared to controls (25). In contrast, studies by Parikh et al. and Van Driest et al. found no significant mortality difference, highlighting heterogeneity in patient populations or study methods (22, 29).

As anticipated, most studies reported a significant increase in both hospital and PICUs length of stay among patients with AKI (21, 23, 25–27, 29, 32). These results correspond to the studies of an adult population (62, 63).

A markedly high rate of kidney function recovery was reported, with figures ranging from 30% to 100% (20, 33). Similarly, studies involving adult cohorts indicate that complete or partial kidney function recovery occurs in approximately 30% of cases across all age groups, whereas the incidence of non-recovery tends to increase with advancing age (61, 64, 82–85). Nonetheless, De Zan et al. observed that 11.8% of pediatric patients with stage III AKI who did not require dialysis continued to exhibit impaired kidney function during follow-up (26). Similar to the findings of De Zan et al., Paudel et al. reported that 5 out of 56 patients with AKI exhibited incomplete recovery, as evidenced by persistently abnormal serum creatinine levels (86). Differences in sample sizes may partially explain this discrepancy; smaller cohorts tend to report higher recovery rates, possibly due to limited statistical power.

Long-term outcomes of AKI remain under-investigated. The majority of studies report favorable kidney function post-AKI. Although Louzada et al., Nawaz et al., and Ashish et al. identified 2, 5, and 10 cases, respectively, that progressed to CKD during follow-up, indicating that long-term kidney sequelae, though infrequent, are nonetheless significant (29, 31, 35). Moreover, there is limited data available on the incidence and outcomes of recurrent AKI in the pediatric population. The only study by Chisavu et al. found that persistent acute kidney disease is an independent risk factor for CKD development (33). Similarly, there is a lack of evidence regarding the long-term impact of AKI experienced during childhood on the progression and severity of CKD in adulthood.

Regarding economic impact, only two studies addressed this aspect: Xu et al. and Sun et al. found that AKI was significantly associated with increased daily and total hospitalization costs (*p* = 0.003), underscoring the financial burden imposed by the condition (25, 37).

### 4.4 Strengths and limitations

This study encounters several limitations. Primarily, several articles met all predefined inclusion criteria but were not captured by our search strategy (67, 87). To preserve methodological consistency and replicability, they were excluded from the final analysis. This outcome was surprising to the authors, considering the high-impact and reputability of the journal in which they were published. It highlights the need to include all the essential keywords in manuscripts to ensure retrieval by focused search methodologies; a requirement that will become increasingly vital with the ever-growing volume of scientific publications. Secondly, the limited number of studies included in the analysis may restrict the extent to which the findings can be generalized. Furthermore, significant heterogeneity was observed, manifesting in varied study designs, diverse outcome measures, and considerable variability in sample sizes, all of which challenge the comparison and synthesis. Finally, the use of only two databases and the absence of EMBASE access may have constrained the overall scope and potentially omitting studies indexed exclusively in subscription-based databases.

Conversely, a notable strength of this systematic review lies in its precise inclusion criteria, which were meticulously designed to ensure a focused investigation of the topic and to reduce bias in study selection. Another advantage is the overall low risk of bias across the included studies, which enhances the reliability of the review's conclusions and supports the formulation of recommendations for future research.

## 5 Conclusions

Prerenal AKI is the predominant type in high-risk pediatric settings such as PICU, post-cardiac surgery, and cases with moderate to severe volume depletion, reflecting patterns seen in adults. However, unlike adults—where multifactorial AKI becomes increasingly frequent with age — most pediatric AKI cases are unifactorial. Although awareness of AKI has improved, long-term follow-up studies remain largely nonexistent, possibly due to the fact that most children recover from AKI without lasting sequelae. However, the potential impact of repeated AKI episodes requires further investigation, with particular attention to the progression to CKD and the development of hypertension.
